# MOSFET Physics-Based Compact Model Mass-Produced: An Artificial Neural Network Approach

**DOI:** 10.3390/mi14020386

**Published:** 2023-02-04

**Authors:** Shijie Huang, Lingfei Wang

**Affiliations:** 1 Key Laboratory of Microelectronics Devices and Integrated Technology, Institute of Microelectronics, Chinese Academy of Sciences, Beijing 100029, China; 2 State Key Laboratory of Fabrication Technologies for Integrated Circuits, Institute of Microelectronics, Chinese Academy of Sciences, Beijing 100029, China; 3 University of Chinese Academy of Sciences, Beijing 101408, China; 4 Peng Cheng Laboratory, Shenzhen 518066, China

**Keywords:** surface potential, MOSFET, artificial neural network, DIBL, compact model

## Abstract

The continued scaling-down of nanoscale semiconductor devices has made it very challenging to obtain analytic surface potential solutions from complex equations in physics, which is the fundamental purpose of the MOSFET compact model. In this work, we proposed a general framework to automatically derive analytical solutions for surface potential in MOSFET, by leveraging the universal approximation power of deep neural networks. Our framework incorporated a physical-relation-neural-network (PRNN) to learn side-by-side from a general-purpose numerical simulator in handling complex equations of mathematical physics, and then instilled the “knowledge’’ from the simulation data into the neural network, so as to generate an accurate closed-form mapping between device parameters and surface potential. Inherently, the surface potential was able to reflect the numerical solution of a two-dimensional (2D) Poisson equation, surpassing the limits of traditional 1D Poisson equation solutions, thus better illustrating the physical characteristics of scaling devices. We obtained promising results in inferring the analytic surface potential of MOSFET, and in applying the derived potential function to the building of 130 nm MOSFET compact models and circuit simulation. Such an efficient framework with accurate prediction of device performances demonstrates its potential in device optimization and circuit design.

## 1. Introduction

Compact models work as a bridge between the fabrication process and circuit design. They are designed to accurately reproduce minute details of device electrical characteristics, which are essential in the design of digital, analog, mixed-signal, and RF-integrated circuits. This requires the model to be accomplished in a manner consistent with the device operation physics, and with a model structure that remains invariant of fabrication process particulars. Several types of compact model for a Mental-Oxide-Silicon Field-Effect transistor (MOSFET) have been developed, including the threshold-voltage($V_{\mathrm{th}}$)-based compact model, the inversion-charge($q_{i}$)-based compact model, and the surface-potential($\phi_{s}$)-based compact model. Among these compact models, the $\phi_{s}$-based compact model has achieved widespread success and is most frequently used in modern circuit simulators for its accurate description of major physical effects, which are responsible for the characteristics of scaled MOSFETs. The expression formulation of $\phi_{s}$ is the key component of $\phi_{s}$-based compact models, and needs to be carefully designed in solving implicit transcendental equations. Aggressive down-scaling of the device and the resultant physical effects have made it very challenging to obtain analytic solutions from complex equations in mathematical physics [1,2]. On the other hand, though numerical solvers [3] can be high-quality alternatives, the non-differentiable solution is unfit for circuit simulation or design optimization; furthermore, a solver may take hours just to solve the equation for a single condition, being computationally intractable even if it can be applied on demand.

Can we free ourselves from challenging theoretic effort and maximally automate the process of building an analytic device surface potential expression that can be applied efficiently in system simulation, design, and optimization? The strength of an artificial neural network (ANN) is considered as one of the effective ways by which to achieve the goal. An ANN endeavors to recognize underlying relationships in a set of data through a process that mimics the way the human brain operates and can adapt to changing input, so the network generates the best possible result without needing to redesign the output criteria. An ANN consists of an input layer of neurons taking the input data, one or two hidden layers of neurons processing the input data, and a final layer of output neurons sending the predicted output, which is compared with the actual output. Based on the error, the parameters (weight and bias) in an ANN are changed and are then fed into the network again to improve the accuracy of prediction. Compared to other machine learning algorithms, an ANN has the ability to learn and adapt to unknown systems, and can fully approximate any complex nonlinear relationship, giving analytical expression of the input and output data, which is essential in the compact model development.

Several studies have been conducted to apply artificial neural networks in building models for MOSFET. In the literature [4,5], the electrical characteristics of MOSFET, including I-V curves and C-V curves, were directly adopted as the training data of the ANN. Two hidden layers were involved in the network and the learning result is achieved. However, with little consideration of the physics of the device’s working principle, the modeling variation is complex in the pure-deep-learning-based model, which is important in device design and optimization. In [6], a deep-learning-assisted MOSFET I-V compact modeling method was proposed. In the method, the ANN acts as a correction term to the traditional BSIM compact model, which increases the compatibility of the classical model with advanced node devices. This method presents a possible prospect for the development of future compact models. However, the direct correction to the I-V curve by the ANN also conceals the important physical information in advanced MOSFETs, which is the optimization and development of scaling devices.

In this work, we propose an innovative artificial neural network (ANN) [7] to obtain the surface potential for compact modeling, and thus, preserve physical information in the subsequent process of building the compact model. The key idea is to use a dedicated numerical simulator [8] (e.g., TCAD) as a “teacher”, and feed its output across highly diverse device data to the “student”, a so-called physical-relation-neural-network (PRNN). The PRNN is a universal approximator that combines general-purpose data fitting with domain-specific physical relations. Therefore, it can effectively mimic the behavior of the teacher, and instill the learned “knowledge” from the solver into the neural network, effectively closing the gap between discrete, numerical simulations and continuous, analytic modeling. We demonstrate the impressive results of our approach in obtaining accurate, analytic surface potential in 130 nm MOS devices. It only requires a simple substitution to generalize to the new parameters of the device, and human intervention involved in different devices/equations [9] is also expected to be minimal. Our framework will be particularly useful in device simulation acceleration, speeding up and coupling the development of micro-device physics-based compact models and device design optimization.

## 2. Method Framework

**Figure 1:**
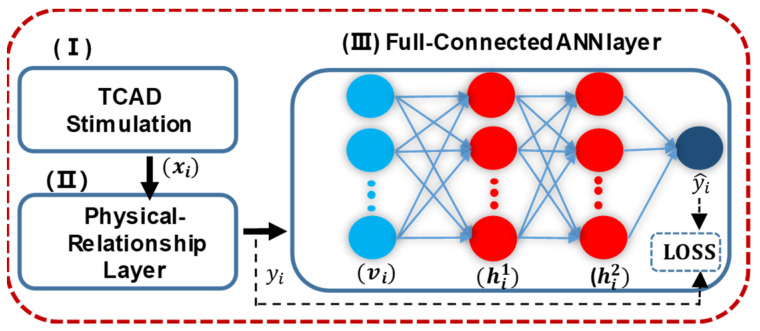
Schematic of the PRNN Framework. Stimulation data from TCAD is firstly transformed at the physical-relationship layer to contain more fundamental relations of physics. Then, the pretreated data is trained by fully-connected artificial neural networks.

The global picture of the proposed framework is shown in Figure 1. First, TCAD [8] software is used to perform the simulation and generate the training data. The data are fed into a physical-relation-neural-network (PRNN), which is composed of both a physical-relation layer to account for basic, low-level physical prior knowledge, and general-purpose fully-connected layers to further capture data nonlinearity. After training and network optimization, the resultant analytic surface potential is further applied to building 130 nm MOSFET semi-classical compact models (e.g., I-V/C-V characteristics) and circuit simulation.

### 2.1. TCAD Simulation

**Figure 2:**
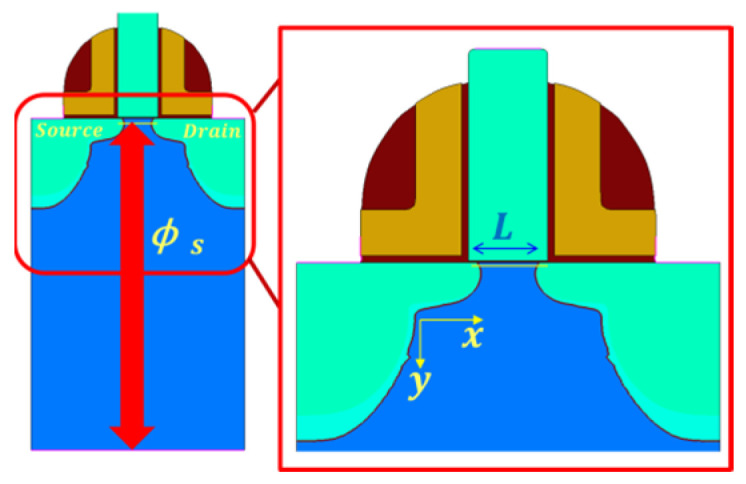
Schematic of the 130 nm MOSFET device with a polysilicon gate stimulated in TCAD. The surface potential is defined as the difference between the substrate electrostatic potential and the electrostatic potential (one nanometer below the channel surface).

The 130 nm node MOSFET is stimulated with the Sentaurus Device TCAD tool, as shown in Figure 2. The simulated training data have two parts: preselected device data/parameter $x_{i}^{\prime }s$ (i for sample index), and corresponding surface potential [10] value $y_{i}^{\prime }s$ computed by TCAD. Each $x_{i}^{\prime }s$ is a *d*-dimensional vector specifying: the thickness of the gate insulation layer ($T_{\mathrm{ox}}$), the gate to source voltage ($V_{\mathrm{gs}}$), the drain to source voltage ($V_{\mathrm{ds}}$), the length of the channel region ($L_{g}$), the temperature (T), the doping concentration in the channel region ($N_{d}$), and channel locations (x) (Table 1 for details). The $x_{i}^{\prime }s$ should cover a diverse range of device/operation conditions to generate a rich training dataset. The simulated potential value $y_{i}$ is the difference between substrate electrostatic potential and electrostatic potential (one nanometer below the channel surface) [11].

### 2.2. Physical-Relationship (PR) Layer

The PR-Layer groups the variables in each $x_{i}$ and applies group-wise transform to account for the desired interaction between parameters; the details are shown in Equation (1), which reflect useful prior knowledge on some simple but fundamental relations of physics [12]. From a learning perspective, incorporating justified variable interactions can effectively reduce sample complexity; i.e., the amount of data needed for training an accurate model [13,14].

(1) $$v_{i}{=[V}_{\mathrm{gs}}{,V}_{\mathrm{ds}}{,N}_{d}{,\;T,\;x,\;e}^{\lambda \frac{x-L}{t_{\mathrm{si}}}}{,e}^{\lambda \frac{-x}{t_{\mathrm{si}}}}{,V}_{\mathrm{ds}}e^{\lambda \frac{-L}{t_{\mathrm{si}}}}{,V}_{\mathrm{bi}}(1-e^{\lambda \frac{-L}{t_{\mathrm{si}}}}{),V}_{\mathrm{gs}}^{2}{,V}_{\mathrm{gs}}-V_{\mathrm{ds}},\log(\frac{L_{\mathrm{Di}}V_{\mathrm{gs}}}{t_{\mathrm{si}}})].\;$$

Here,$\;v_{i}\in {\mathbb{R}}^{12\times 1}$, $\lambda =\sqrt{\frac{\varepsilon_{\mathrm{ox}}t_{\mathrm{si}}}{T_{\mathrm{ox}}\varepsilon_{\mathrm{si}}}}$, $L_{\mathrm{Di}}=\sqrt{\frac{\varepsilon_{\mathrm{si}}v_{t}}{{\mathrm{qn}}_{i}}}$ is the intrinsic Debye length, $V_{b}{=v}_{t}\ln(\frac{N_{\mathrm{sd}}N_{d}}{n_{i}^{2}})$ is the build-in potential at the source/drain terminal. Min-Max normalization is then adopted to standardize the data $v_{i}$ [15].

### 2.3. Fully-Connected (FC) Layer

In FC-Layers, all neurons in one layer will be fully connected to all neurons in the next layer. These are general-purpose network components, and serve as a nice complement to the PR-Layer in capturing complex nonlinear relations [16] between the device data and surface potential. As shown in Figure 1, we cascade two FC-layers right after the PR-layer, with 64 and 32 neurons, respectively, each activated by the sigmoid function. The two FC-layers are as follows [17].

(2) $$h_{i}^{1}=\sigma \left(W_{1}^{T}\cdot {\;v}_{i}{+b}_{1}\right),$$

(3) $$h_{i}^{2}=\sigma \left(W_{2}^{T}\cdot {\;h}_{i}^{1}{+b}_{2}\right).$$

Here, $h_{i}^{1}\in {\mathbb{R}}^{64\times 1},\;h_{i}^{2}\in {\mathbb{R}}^{32\times 1}\;$are the neurons in the hidden layer, $W_{1}\in {\mathbb{R}}^{12\times 64}$, $W_{2}\in {\mathbb{R}}^{64\times 32}$ are the coefficient weight term matrices of the two FC-layers, $b_{1}\in {\mathbb{R}}^{64\times 1}$ and $b_{2}\in {\mathbb{R}}^{32\times 1}$ are the bias term, and σ(·) is an entry-wise sigmoid function [18].

(4) $$\sigma \left(u\right)=\frac{1}{1+\exp\left(-u\right)}.$$

The sigmoid function is well bounded and allows for efficient computation of the gradient. Finally, the predicted surface potential associated with each $x_{i}^{\prime }$ is computed by

(5) $$\hat{y_{i}}{=w}^{T}\cdot {\;h}_{i}^{2}+b.$$

Here, $w\in {\mathbb{R}}^{32\times 1}$ and b∈ℝ are model coefficients and bias. $\hat{y_{i}}\in \mathbb{R}$ is the predicted value of the ANN. Mean-Squared-Error (MSE) $\sum {\left(y_{i}-\hat{y_{i}}\right)}^{2}/n$ [19,20] is used as the loss function, which is iteratively minimized with stochastic gradient descent [21].

Upon the completion of the training process, the analytic expression of the surface potential can be written as:

(6) $$\hat{y_{i}}(\mathrm{or}\;\phi_{s}{)=w}^{T}\;\sigma \left(W_{2}^{T}\cdot \sigma \left(W_{1}^{T}\cdot {\mathrm{PR}(x}_{i}{)+b}_{1}\right){+b}_{2}\right)+b.\;$$

which is a concise model and can be efficiently evaluated.

### 2.4. Surface Potential Written in Verilog-A

To further verify the applicability of the framework in circuit simulation, the trained artificial neural network should be transformed into the form of Verilog-A [22], which is the commonly used hardware description language for MOSFET and other electronic components. Verilog-A is the analogy subset of Verilog-AMS [23], originally intended for modeling the behavior of analog and mixed-signal systems. Despite significant initial resistance, Verilog-A has emerged as the de facto stand language for defining and distributing compact models. In 2004, constructs explicitly for the purpose of compact modeling were added. Considering the incompatibility of matrix calculations in Verilog-A language, an automation script (Python [24], for example) is adopted to accelerate the transform process. Two steps are divided to realize this purpose. First, the value of the parameters in each layer should be entered into Verilog-A. In the framework, these parameters include physical parameters (for example, vacuum dielectric constant of silicon [25] $\varepsilon_{\mathrm{si}}$, Planck constant k, and Unit charge constant q) used in PRNN, Min and Max value for normalization, bias term $b_{i}$, and weights term $w_{i}$. Table 2 (I) shows the pseudo code [26] for inputting $w_{i}$. The value of $w_{i}$ is read from the saved txt file and written to Verilog-A by intermediate variable a. Then, in the second step, the forward propagation process of the artificial neural network is realized in Verilog-A. In this step, the implementation of the calculation process in the framework includes the data normalization, neurons ($h_{i}$) in the hidden layers and denormalization of data at output data $\hat{y_{i}}$. Table 2 (II) shows the pseudo code for transforming $h_{i}^{2}$. The calculation of weight and bias terms between each neural is conducted following Equations (3) and (4).

### 2.5. Establishing the Compact Model

After obtaining the analytical surface potential expression, the related compact model for MOSFET can be built. In this work, a semi-classical compact model is developed based on the combination of trained surface potential expression and the classical compact model. The main equations are shown as follows: [27,28,29,30,31]

(7) $$I_{\mathrm{ds}}{=u}_{\mathrm{eff}}C_{\mathrm{ox}}\frac{W}{L}\left(V_{\mathrm{gf}}\left(\phi s_{d}-\phi s_{s}\right)-0.5\left(\phi s_{d}^{2}-\phi s_{s}^{2}\right)\right).\;$$

(8) $$u_{\mathrm{eff}}=\frac{u_{0}}{1+{\mathrm{MUE}*E}_{\mathrm{eff}}{}^{\mathrm{THEMU}}+\mathrm{CS}{\left(\frac{q_{d}}{q_{d}+q_{i}}\right)}^{2}}$$

(9) $$V_{\mathrm{gf}}=\mathrm{Log}\left[1+e^{\left(V_{\mathrm{gs}}-V_{\mathrm{fb}}\right)*\mathrm{Sl}}\right]/\mathrm{Sl}$$

(10) $$\phi s_{s}=\phi s\left[V_{\mathrm{gs}},V_{\mathrm{ds}},T,\ldots ,x]-\phi s[0,0,T,\ldots ,x\right]$$

(11) $$\phi s_{d}=\phi s_{s}+V_{\mathrm{dsx}}$$

(12) $$V_{\mathrm{dsx}}={\mathrm{gV}}_{\mathrm{ds}}{\left(1+{\frac{{\mathrm{gV}}_{\mathrm{ds}}}{V_{\mathrm{dsat}}}}^{m}\right)}^{-\frac{1}{m}}$$

Here, $u_{\mathrm{eff}}$ [29] is carrier mobility, $u_{0}$ is carrier mobility at a low electrical field, $q_{d}$ is the normalized charge of the depletion region, $q_{i}$ is the normalized charge of the inversion region, (MUE,THEMU) is the fitting parameters accounting for the mobility degradation [32] caused by the surface roughness and phonon scattering. Coulomb scattering is introduced using the parameter CS. $E_{\mathrm{eff}}$ denotes the effective vertical field at the potential midpoint. $V_{\mathrm{gf}}$ [33] accounts for the subthreshold region with parameter Sl acting as the correction parameter to the subthreshold swing. $\phi s_{s}$ is the surface potential obtained from the ANN at x = 0.01 um. $V_{\mathrm{dsat}}$ [31] is the saturation voltage with m acting as a fitting parameter.

The gate capacitance model is also necessary in circuit simulation and can be calculated as follows [29,34,35,36]:

(13) $$Q_{g}={W*C}_{\mathrm{ox}}\int_{0}^{L}\left(V_{g}-V_{\mathrm{fb}}-\phi s\left[V_{\mathrm{gs}},V_{\mathrm{ds}},T,\ldots ,x\right]\right)\mathrm{dx}$$

(14) $$Q_{d}={W*C}_{\mathrm{ox}}\int_{0}^{L}q_{i}\frac{y}{L}\mathrm{dx}$$

(15) $$Q_{S}={W*C}_{\mathrm{ox}}\int_{0}^{L}q_{i}\left(1-\frac{y}{L}\right)\mathrm{dx}$$

(16) $$C_{\mathrm{mn}}=-\frac{\partial Q_{m}}{\partial V_{n}},m\neq n\;;C_{\mathrm{mm}}=\frac{\partial Q_{m}}{\partial V_{m}}$$

## 3. Method Validation and Discussion

**Figure 3:**
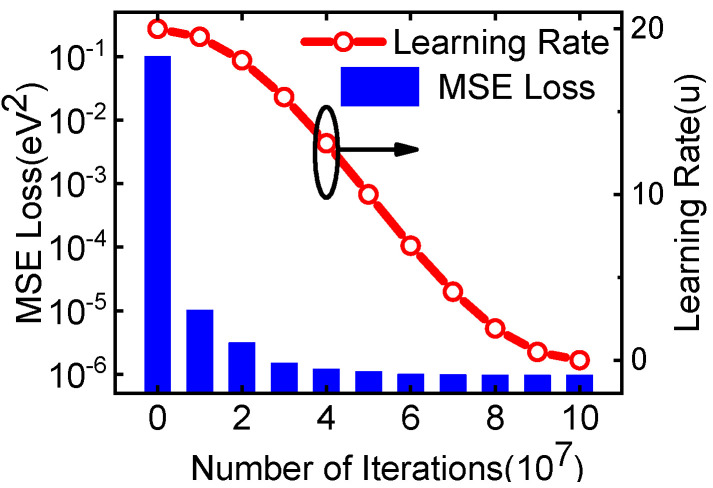
Mean Square Error (MSE) and learning rate with training iterations.

We evaluated the proposed framework by computing the surface potential in 130 nm MOSFET. We generated 540,000 training samples and 100,000 testing samples by TCAD simulation. The *d*-dimensional device data $x_{i}^{\prime }s$ were generated by randomly sampling each variable from their feasible domains. The evaluation results are reported in Figure 3. The left coordinate in Figure 3 shows the relationship between the MSE testing error and the training iteration process. It can be found that during the training process, the MSE loss decreases and stabilizes at $9.58\times 10^{-7}$, which is in millivolts, indicating a highly accurate result. The learning rate is an important hyper-parameter in the training process. A large learning rate promotes the rapid reduction of learning errors, while a small learning rate contributes to the convergence of the model. In this work, the learning rate is set to gradually decrease from 2 × 10^−5^ to 1 × 10^−8^ using the cosine annealing algorithm (a half cycle is adopted) during the training process, as the right coordinate in Figure 3 shows.

**Figure 4:**
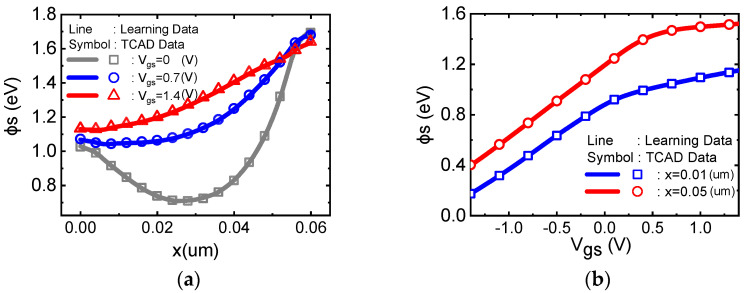
An excellent match between our PRNN model (lines) and the TCAD numerical simulation (symbols) for (**a**) the surface potential $\phi_{s}$ along the device channel against different gate voltages and (**b**) the surface potential against the gate voltage at the source/drain terminal.

Figure 4a plots the 2D surface potential along the device channel. At a low gate voltage, the surface potential in the middle of the channel is determined by gate−channel work function differences. The surface potential of the drain and source terminal are raised by the PN junction[37], which is induced by different doping types of channel and source/drain terminals, and are hardly affected by $V_{\mathrm{gs}}$. When $V_{\mathrm{gs}}$ increases from 0 (V) to 1.4 (V), the surface potential in the channel increases due to the electrical field induced by gate voltage $V_{\mathrm{gs}}$, while the potential at the drain/source terminal stays almost fixed. Thus, the minimum surface potential moves from the middle of the channel to the source terminal, which is a challenging feature that a traditional 1D Poisson equation [38] solution fails to capture. We found an excellent match between our model predictions and the TCAD simulation. Figure 4b plots the surface potential at the source and drain terminal versus the gate voltage, respectively. Excellent agreement is achieved between the TCAD simulation result and the PRNN result. When the gate voltage increases, the surface potential at the drain/source terminal increases first and then gradually saturates, which is consistent with previous reports [8].

**Figure 5:**
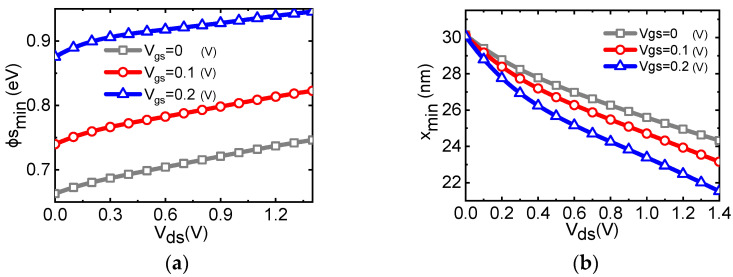
(**a**) The lowest potential ($\phi s_{\min}$) w.r.t. drain voltage changes and (**b**) the lowest surface potential location ($x_{\min}$) andThe DIBL effect is observed for the shift of $x_{\min}$ and $\phi s_{\min}$ with $V_{\mathrm{ds}}$.

Figure 5 plots the patterns of the minimum surface potential ($\phi s_{\min}$), as well as the location of the minimum potential (x_min_) along the channel; both w.r.t. drain voltage. It can be seen that the two patterns show an opposite trend; with the increase in drain terminal voltage, x_min_ moves to the source terminal and the $\phi s_{\min}$ is increased, which is called the drain-induced barrier lowing (DIBL) effect. $\phi s_{\min}$ decides the threshold voltage of the device according to Equation (8) [39].

(17) $$\Delta V_{t}=-\eta \Delta \phi s_{\min}=-{\sigma V}_{\mathrm{ds}}.\;$$

Here, η is the ideality factor and is assumed to be independent of bias condition. σ is a physical parameter that reflects the influence of $V_{\mathrm{ds}}$ to threshold voltage. The DIBL effect causes an excess injection of the charge carrier into the channel and gives rise to an increased subthreshold current [40]. The overlaps of the gate depletion zone with the source/drain depletion zone share its depletion charge [41], and the shared charge is balanced by a counter charge distributed between the gate electrode and the source and drain contacts, which brings a shift in threshold voltage. With the introduction of the PRNN, analysis of the device against the DIBL effect could be conducted in a facile way, and thus facilitates the optimization of device performance.

**Figure 6:**
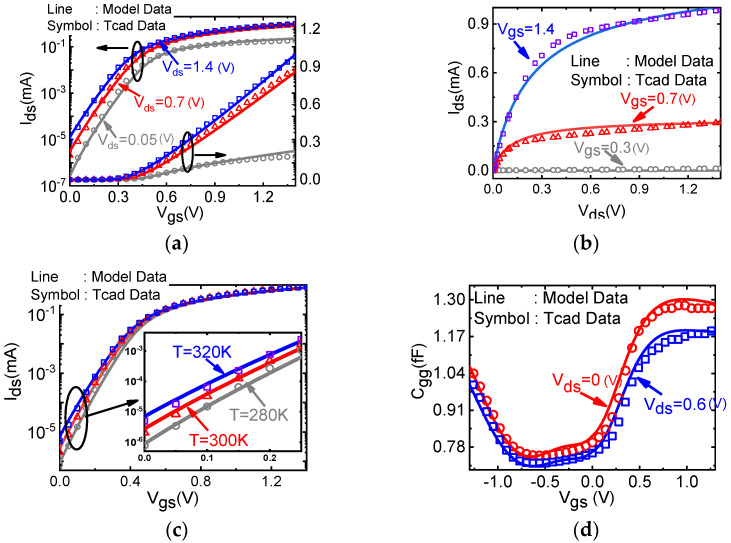
A good match between our MOSFET compact model (line) and the TCAD solution data (symbol) for (**a**) output characteristics, (**b**) transfer (both in conventional and logarithmic coordinates) characteristics, (**c**) transfer characteristics with different temperature, and (**d**) gate capacitance to gate voltage.

Figure 6 shows the comparison of the developed surface potential based semi-classical compact and TCAD simulation results. Good agreement is achieved between the n-type transfer characteristic curve against different drain voltages (a), the output characteristic curve (b), the transfer characteristic curve against different operation temperatures [42], and (d) small signals gate capacitance $C_{\mathrm{gg}}$. It shows that our model can well describe the device performance.

**Figure 7:**
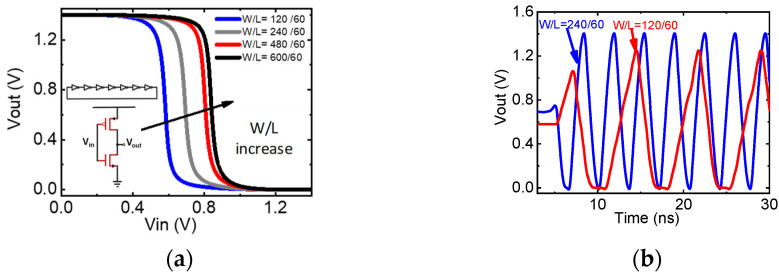
(**a**) The circuit diagram of a ring oscillator composed of seven-stage inverters connected in series; the inverter structure is shown at the bottom. The relation between V-out and V-in for the inverter: with the size of the p-type MOSFET pull up transistor increasing, the pull up driver capability increases as well, causing the curves to shift to the right. (**b**) Transient simulation results of the ring oscillator: with the size of the p-type MOSFET transistor decreasing, the frequency of the oscillator is reduced and saturation appears at the lowest point of oscillation.

To further verify the applicability of the framework to modern microelectronic circuit design, the proposed compact model was transformed into Verilog-A and circuits simulation was conducted by including the MOSFET devices as new active components of the circuit simulator, as Figure 7 shows. A ring oscillator circuit with seven-stage inverters was connected in series to generate oscillation [43]. Figure 7a shows the Vout-vs-Vin curves of the inverter. When the size of the p-type MOSFET increases, the driver capability of the pull up increases, and thus, the Vout-vs-Vin curves shift to the right. Figure 7b shows the transient simulation results. When the size of the p-type MOSFET transistor decreases, the frequency of the oscillator decreases as well, and saturation appears at the lowest point of the oscillation. These simulation results clearly demonstrate the applicability and usefulness of our framework in circuit simulation applications.

Compared to the classical compact model method [29,44], the proposed framework avoids the numerical iteration process [14] in solving the surface potential expression, and thus, saves a great deal of effort in model design. Considering that most physical effects caused by device scaling directly act on surface potential, the proposed framework can better achieve underlying physic scaling compared to those works that directly train electrical properties of a device that is incompatible with model variation. Furthermore, in the literature [6], due to training data being obtained through the subtraction of device electrical properties and classical compact model output, the ANN acts as a correction term to existing models and contains scarcely underlying physical information. In contrast, the training data of the surface potential in this framework is obtained from TCAD simulators, which are based on equations of mathematical physics and reflect the physical relationship between device parameters and surface potential, thus containing more systematic physical information of the device.

## 4. Conclusions

We exploited the universal approximation power of artificial neural networks in learning from large amounts of simulation data to generate accurate, generalizable MOSFET compact models in a highly automated manner. Impressive results were reported in building the analytic surface potential of a 130 nm MOSFET, which proved to be of benefit in device optimization. Furthermore, our work reveals the great potential of modern artificial intelligence techniques in boosting microelectronic research. The accurate, generalizable, and automated compact model development not only reduces the gap between theory and computing, but it is also expected to bring new vigor to vast landscapes in design, simulation, and the optimization of very large-scale circuit systems.

## Figures and Tables

**Table 1:** Features of stimulated device data $x_{i}$.

Term	Min Value	Max Value	Std
$V_{\mathrm{gs}}$ (V)	−1.4	1.4	0.81
$V_{\mathrm{ds}}$ (V)	0	1.4	0.418
T (K)	275	300	14.14
x (nm)	0	70	48.59
L (nm)	50	70	7.07
$N_{d}\;({\mathrm{cm}}^{2}$)	1 × 10^17^	1 × 10^19^	3.7 × 10^18^
$\phi_{s}$ (eV)	−0.08438	2.405	0.49443

**Table 2:** The pseudo code for transforming the ANN to Verilog-A language.

**(I) Parameter Input** **for** $\;w_{i}$
for $w_{i}$ in framework with file.open($w_{i}$_value.txt) as f1:
for line in f1.readlines():
line_list = line.split() for j in range(len(line_list)):
a = “parameter real $w_{i}$” + str(i) + ’ = ‘+str(line_list[j]) + ’;’
f.write(a) i = i + 1 i = 1
**(II) Forward Propagation Realization for** $\;h_{2}$
for i in range(1,len(hidden layer 2)):
a = ‘$h_{2}$’ + str(i) + ’ = ‘ + ’1/(1 + exp(-(‘
for j in range(1,len(hidden layer 1)):
a = a + ’$w_{2}$’ + str(i) + str(j) + ’*$h_{1}$’ + str(j) + ’ + ’
a = a + ’$b_{2}$’ + str(i) + ’;’ f.write(a)

## Data Availability

The data presented in this study are available on request from the corresponding author.
